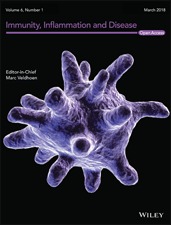# Issue Information

**DOI:** 10.1002/iid3.193

**Published:** 2018-02-19

## Abstract